# Salmagundi

**Published:** 1887-05

**Authors:** 


### SALMAGUNDI.

EDITED BY E. G. BETTY, D.D.S.

W. E. SCOTT of Springfield, Mo., says that a closely fitting copper wire worn constantly around the neck will often cure obstinate cases of facial neuralgia.

A LOS ANGELES surgeon recently prescribed some rectal suppositories for an old soldier. The next norning the doctor called on him and asked him how he was. He answered: "First rate, doctor; but them catridges was mighty hard to swallow. I got 'em down, though."—*Exchange.*

A FASHIONABLE London doctor has startled folks by asserting that tight lacing is a public benefit. He takes the unassailable ground that it causes the fools among women to die young.—*Clipping.*

---

THE conductor of the DENTAL ECLECTIC publishes a letter in the March number from Bishop Elder of Cincinnati. We would suggest that the reverend contributor is an Archbishop, a grade or two higher than our worthy friend of the ECLECTIC makes him.

---

THE LONDON *Lancet* defines "moderate drinking" as that which consists with a clean tongue, a good appetite, a slow pulse, a cool skin, a clear head, a steady hand, good walking power, and light, refreshing sleep, and asserts that "odd glasses of beer and spirits in a forenoon do not come within the range of moderate drinking." Paste this in your hat.

---

TREATMENT OF SCIATICA.—Dr. Metcalf, of New York, says that no prescription for sciatica has ever equaled in efficacy the following: ℞. Tinct. aconit. rad., tinct. colchic. sem., tinct. belladonna, aa ʒ j. M. Sig.: Dose, six drops every six hours. He also uses triturate tablets, each containing three drops of the following: Tincture of anconite root, tincture of seeds of colchicum, tincture of belladonna, tincture of actea racemosa—equal parts by volume. Dose, one every four or eight hours.—*Medical Progress.*

---

MEDICAL LITERATURE.—Under this general title Mr. Howard Challen, of 150 Nassau Street, New York, is preparing for publication the lists of books issued by medical book publishers in uniform octavo style. It will have an index of all medical, surgical, dental, and sanitary books, arranged alphabetically, under author, subject, and title, and all periodicals, with subscription price relating thereto, making it indispensable to book buyers, for whom it is especially intended.

Authors and book-buyers will obtain further information by addressing the publisher.—*Exchange.*

NITROUS PROTOXIDE A DANGEROUS ANÆSTHETIC.—M. Grehaut stated before the Societe de Biologie that nitrous protoxide does not act as an anæsthetic, but as an asphyxiating agent. Dentists happily describe its effect by saying that their patients "virent," which means that they turn blue. M. Grehaut believes that deaths from laughing-gas are few because it is used for short operations, but he affirms that it is an essentially dangerous anæsthetic, and that its indiscriminate use ought to be forbidden. M. Lafont has ascertained that accidents have occurred after it has been administered.

---

MEDICAL and other professional men often break down from their inability to keep a regular time for meals. An eminent doctor says (*Scientific American*): "Being often out for many hours, and becoming too exhausted to digest a full meal when at length able to get it, I conceived a plan which answered admirably well, and which other doctors have gladly adopted. I provided myself with a small bottle of lime water, which I added to a glass of milk when passing a dairy shop, or I put a small flask of the mixture in my pocket. A water biscuit with this will keep a man harmless on a long fast and enable him to digest a meal when he can obtain it."

---

NEW PEPSIN PREPARATION.—A new pepsin has been placed in the market by the well-known manufacturing house of William F. Kidder & Co., of New York, under the name of Kidder's Crust Pepsin. It is in scales, and is non-hygroscopic under ordinary circumstances. In its power of dissolving albuminoids, animal tissues, or diphtheritic membrane, this form of pepsin, it is claimed, is twice the strength of any of the ordinary makes of pepsin, as shown by the albumen test. It is quite uniform in composition, free from salt, sugar of milk, and peptones. By actual test, one part was found to dissolve from twelve hundred to fifteen hundred parts of coagulated egg-albumen. The chemist of the Brooklyn Board of Health states that when tested with the beef-process it digests double the quantity that any other pepsin in the market will. Saccharated pepsin, U.S.P., can be made by adding nineteen grains of sugar of milk to one grain of Kidder's pepsin. In this manner of preparing saccharated pepsin it is said that only one-sixth as much is used of this form of pepsin as would be required of other makes. As the price will not be higher than that of other kinds, it is evident that this will be a cheap as well as an efficient preparation.—*Exchanye.*

---

THE pictures of Washington in his old age seem to caricature the lower part of his face. Even that magnificent portrait of Stuart's, which hangs in the east room of the White House, pictures the mouth and chin as if when the great man sat for the artist he had just put in each cheek a fresh supply of that comfort which is contraband to habits of cleanliness and good taste. The truth about this expression in these portraits of Washington is that at that time of life he was obliged to use false teeth, and they gave him a great deal of trouble. The set he most commonly wore were made for him by a Swiss artisan in New York. They are now preserved in the museum of the Baltimore College of Dentistry. The material used in them was hippopotamus ivory. The lower plate was made of one solid piece, teeth and plate being carved together. The upper plate required greater skill, and was made with the plate separate, and the teeth riveted to it with fine gold rivets. Although plaster casts of Washington's mouth were made, it was impossible to carve the plates precisely as they should be to fit. They gave him great pain at times, but by gradual carving they finally were adapted to the irregular surface of his mandibles. It was his habit to remove his teeth on retiring at night, and in the course of two years, by the moisture of his mouth and the regular nightly cleansing, the upper plate cracked and split, so that one day the father of his country felt the roof of his mouth cleft in twain. Had it not been for the delicate springs inserted at the hinges of these teeth to make them cling tightly to his jaws, in the absence of that perfect suction which the modern dentists secure, the teeth would have fallen out. Washington sent them to a jeweler at Alexandria for repairs. Thin strips of steel were riveted across wires on each piece of the ivory plate, and the old gentleman wore this amended set of his teeth to his death.—*Clippinq.*

